# Ultrastructural study of closed macular hole- preliminary application of a novel high magnification module combining with OCT

**DOI:** 10.1186/s12886-021-01801-0

**Published:** 2021-03-22

**Authors:** Chang-Yu Qiu, Yuan-Yuan Shi, Hong-Wei Zhao, Chuang Nie, Ming-Xia Dong, Huai-Qiang Zhang, Jun Zhao, Qian-Qian Xu, Fei-Long Song, Xiao-Hua Guo, Lin Shi, Chang-Ying Liu, Yu-Bo Gong, Ling Luo

**Affiliations:** 1 Department of Ophthalmology, Strategic Support Force Medical Center, An xiang bei 9#, Beijing, China; 2 Tangshan Ophthalmological Hospital, Tangshan City, Hebei Province China

**Keywords:** Macular hole, Closure mechanism, High magnification module, OCT, Microstructure, Macular displacement

## Abstract

**Background:**

As a novel high magnification module (HMM) combining with OCT (OCT-HMM) is able to detect the microstructure of retina, we apply it to explore the ultrastructure of the macula after closure of the idiopathic macular hole (IMH) by surgery.

**Methods:**

This is an observational case series study in which patients with full-thickness IMHs who had undergone successful macular closure by vitrectomy and internal limiting membrane peeling and healthy subjects were recruited. After comprehensive ophthalmic examinations, the images of macular area were obtained and collected by professional operators using OCT-HMM. Then images were independently analyzed by 4 masked vitreoretinal specialists.

**Results:**

A total of 24 IMH eyes and 42 healthy eyes were examined. HMM images were obtained in 10 IMH eyes. Among them, 4 eyes whose macula closed completely with recovery of photoreceptor layer presented a dark arc nasal to the fovea, oriented to the optic, and the notch of arc faced temporally. Six eyes in which the macula closed incompletely with photoreceptor cells loss revealed a dark ring with uneven bright spots inside. The other 14 eyes failed to obtain clear images by OCT-HMM. The contra lateral eyes of the patients and the healthy subjects’ eyes succeeded to obtain the HMM images which displayed evenly grey background thickly covered with tiny bright dots that was in similar size and evenly and widely distributed and there no dark arc or ring. OCT B-scan and IR images could be acquired in all of the IMH and healthy eyes.

**Conclusion:**

The preliminary application of HMM has supplied us a brand-new insight into the microstructure of closed IMH. A dark arc sign could be detected with OCT-HMM in the macula which was functionally closed after surgery that was probably the healing mark on a microstructure photoreceptors level. Its existence and shape indicated that the functional closure followed by a retinal displacement mainly horizontally from temporal side to nasal side but not symmetric centripetally.

## Background

Macular hole (MH) is a common ophthalmic disease. There are three kinds of MH, idiopathic MH (IMH), traumatic MH and high myopia MH [1]. The diagnosis, treatment, prognosis and pathogenesis of MH have been closely related to the innovation and progression of examination methods [2, 3]. The rapid development of modern science and technology provides very important means for the development and progression of modern medicine, and makes indelible contribution to the health of human beings [4, 5]. In turn, the need of clinical and basic research brings infinite power and source for the development of science and technology. Since optical coherence tomography (OCT) was first applied clinically in 1991 [6], and first applied in macular disease in 1995 [7], the development of OCT and the researches on macular diseases have been more and more refined. At present, benefiting from its high resolution and powerful data processing capacity, OCT cannot only clearly display the subtle structure of different layers of retina, but also noninvasive dynamic observation and measurement of retinal vessels. Moreover, it can also distinguish the tissues during the operation in real time [8]. However, it is not satisfactory. Researchers hope to improve the technology to reach cell level. Recently, researchers of Heidelberg Engineering Ltd. have achieved a novel system which seems like seeing down to the photoreceptor level. (Investigate the retina at the microstructural level). It is called High Magnification Module (HMM). So far, there is few relevant literature report on clinical application of this product [9–11]. Here’s a preliminary application of HMM in healthy subjects and post-operation MH patients.

## Methods

### Ethical approval

The current study was conducted on the basis of Declaration of Helsinki principles and was approved by institutional review board of The PLA Strategic Support Force Characteristic Medical Center and Tangshan Ophthalmological Hospital. Written informed consent was obtained from all participants.

### Subjects

The study was designed as a prospective, observational case series. From May 23th, to May 29th in 2019, and from Aug 6th, to Aug 13th in 2020, and from Nov 16th, to Nov 30th in 2020, 22 full-thickness IMH patients (24 IMH eyes) who had undergone successful micro-incision vitrectomy surgery (MIVS) and internal limiting membrane (ILM) peeling (5 eyes combining with inverted flap technique) in our department and Tangshan Ophthalmological Hospital were recruited. There were 15 women and 7 men with a mean age of 67.2 ± 5.1 years. At the same time, we also enrolled 11 healthy subjects who were age equivalent including 7 women and 4 men with a mean age of 64.5 ± 4.2 years. In all, there were 24 postoperative IMH eyes and 42 healthy eyes.

Each patient had comprehensive ophthalmic examinations including measurements of best-corrected visual acuity (BCVA), non-contact intraocular pressure, slit-lamp examinations, fundus photography and spectral-domain OCT (SD-OCT; HRA Heidelberg, Heidelberg, Germany). Lens status was phakic in healthy eyes and pseudophakic in all of the IMH eyes. And the lenses of healthy eyes were only slightly opacity. There were no obvious vitreous opacity. The fundus could be seen clearly, and the OCT B-scan images could be clearly obtained.

The medical records of IMH patients were reviewed and basic data were obtained from all subjects including medical history. All Patients received 23- or 25-gauge transconjunctival sutureless (MIVS, performed by three surgeons (Dr. CY Qiu, MX Dong, HQ Zhang). All surgeries were performed after administration of retrobulbar anesthesia. First, the trocar and infusion cannula were inserted. Then 3.0 mm phacoemulsification with implantation of intraocular lens was performed in all cases. And then 23- or 25-gauge MIVS was performed using the Alcon Constellation vitrectomy system (Ft Worth, TX, USA). Core and peripheral vitrectomy were performed. Indocyanine green staining was used in internal limiting membrane peeling in all cases. Inverted flap technique was used in five eyes. Fluid–air exchange with a soft-tip extrusion needle and active suction was used to drain posterior pole fluid. Nonexpansile mixtures of perfluoropropane (C3F8) were used in 7 cases. Three eyes were filled with silicon oil which had been removed at the time of study. The other 14 eyes were filled with steriled air at the end of the surgery. At last, TobreDex eye oint were placed on the surface of the eye followed by a patch. Patients were instructed in face-down post-operative positioning for 7 days.

All of the participants had no history of glaucoma, other fundus diseases, eye trauma, high myopia (over -5D) or diabetes. The basic information of IMH eyes are shown in Table 1.

**Table 1:** Clinical characteristics of IMH patients

Case	Gender	Age(y)	Eye	Diameter of hole (μm)	Follow-up time(m)	Lens status	BCVA (LogMAR)	Postoperative Macula status	HMM image
Case1	F	74	right	429	63	IOL	0.1	closed with PRL	clear with dark arc
Case2	F	72	left	603	60	IOL	1.1	closed with PRL	clear with dark arc
Case3	F	75	right	450	64	IOL	0.6	closed with PRL	clear with dark arc
Case4	M	70	right	363	4	IOL	0.1	closed with PRL	clear with dark arc
Case5	F	70	left	476	48	IOL	0.7	closed without PRL	clear with dark ring
Case6	M	78	left	1060	21	IOL	1.6	closed without PRL	clear with dark ring
Case7	M	71	left	636	9	IOL	0.2	closed without PRL	clear with dark ring
Case8	F	64	left	604	6	IOL	1.3	closed without PRL	clear with dark ring
Case9	F	66	right	739	8	IOL	0.5	closed without PRL	clear with dark ring
Case10	M	67	right	519	7	IOL	0.4	closed without PRL	clear with dark ring
Case11	M	63	right	445	17	IOL	0.5	closed with PRL	unclear
Case12	F	61	right	1130	17	IOL	1.0	closed with PRL	unclear
Case13	F	77	right	298	13	IOL	0.6	closed with PRL	unclear
Case14	F	69	left	361	12	IOL	0.8	closed with PRL	unclear
Case15	F	63	left	690	41	IOL	0.7	closed with PRL	unclear
Case16	F	63	right	473	2	IOL	0.7	closed with PRL	unclear
Case17	F	71	right	377	7	IOL	0.4	closed with PRL	unclear
Case18	F	60	right	341	5	IOL	0.2	closed with PRL	unclear
Case19	F	50	left	613	3	IOL	0.8	closed with PRL	unclear
Case20	F	60	left	412	6	IOL	0.2	closed with PRL	unclear
Case21	M	65	right	641	8	IOL	0.8	closed with PRL	no image
Case22	M	66	right	512	6	IOL	0.6	closed with PRL	no image
Case23	F	64	left	396	7	IOL	0.4	closed with PRL	no image
Case24	M	65	left	657	4	IOL	0.8	closed with PRL	no image

*Abbreviations*: *IMH* Idiopathic macular hole, *F* Female, *M* Male, *BCVA* Best corrected visual acuity, M Month, *PRL* Photoreceptor layer, *HMM* High magnification module

### Instrument and technology

Spectralis High Magnification Module (HMM) has been recently introduced by Heidelberg Engineering, which is a specific objective lens attachment for the company’s Spectralis confocal scanning laser ophthalmoscope, a multimodal imaging platform optimized for the posterior segment (www.HeidelbergEngineering.com). First, the Spectralis provides the flexibility to identify a region of interest with the standard 30° field of view, and then the HMM magnify it to obtain high-resolution 8° × 8° infrared reflectance (IR) images. The resolution power of HMM reaches 1.5 μm. It allows to resolve retinal microstructures, especially the photoreceptor mosaic in vivo without pupil dilation which is not available in a traditional clinical setting [9–11].

### Manipulation of HMM

The instrument was manipulated according to the protocol (www.HeidelbergEngineering.com). Briefly, before scanning, make sure that the subject properly be seated and positioned, and was able to see the fixation light. All patients are presented normal size (smaller than 2.5 mm in diameter). As the pupil size was suggested 1.5 mm [9–11], we kept the examination room bright to further minimal the pupil size smaller than 2.0 mm. Then these steps were followed: disable the auto-brightness option before starting the acquisition to ensure optimal illumination can be achieved; acquire a 30-degree IR image to assess the overall quality (illumination, signal, etc.); optimize the focus, and identify regions of interest; attach the HMM lens and adjust the focus to match the previous focus setting. Then the high-resolution 8° × 8° infrared reflectance (IR) images of interested region was acquired. Zoomed in the field of interest to optimize the amount of discernible retina details. Usually, it spent 5 min per eye: acquiring a 30-degree IR image of one eye usually took less than 1 minute; installing specific objective lens took less than 20 s; adjusting the focus and gaining image of interest zone may take 1 to 2 min. The time varied with different patients’ cooperation and refractive medium. All of the HMM images were scanned and collected by the professional operators (FLS, CYL). Afterwards, 4 vitreoretinal professionals individually analyze the images, and then discussed and interpreted the images together.

## Results

**Fig. 1:**
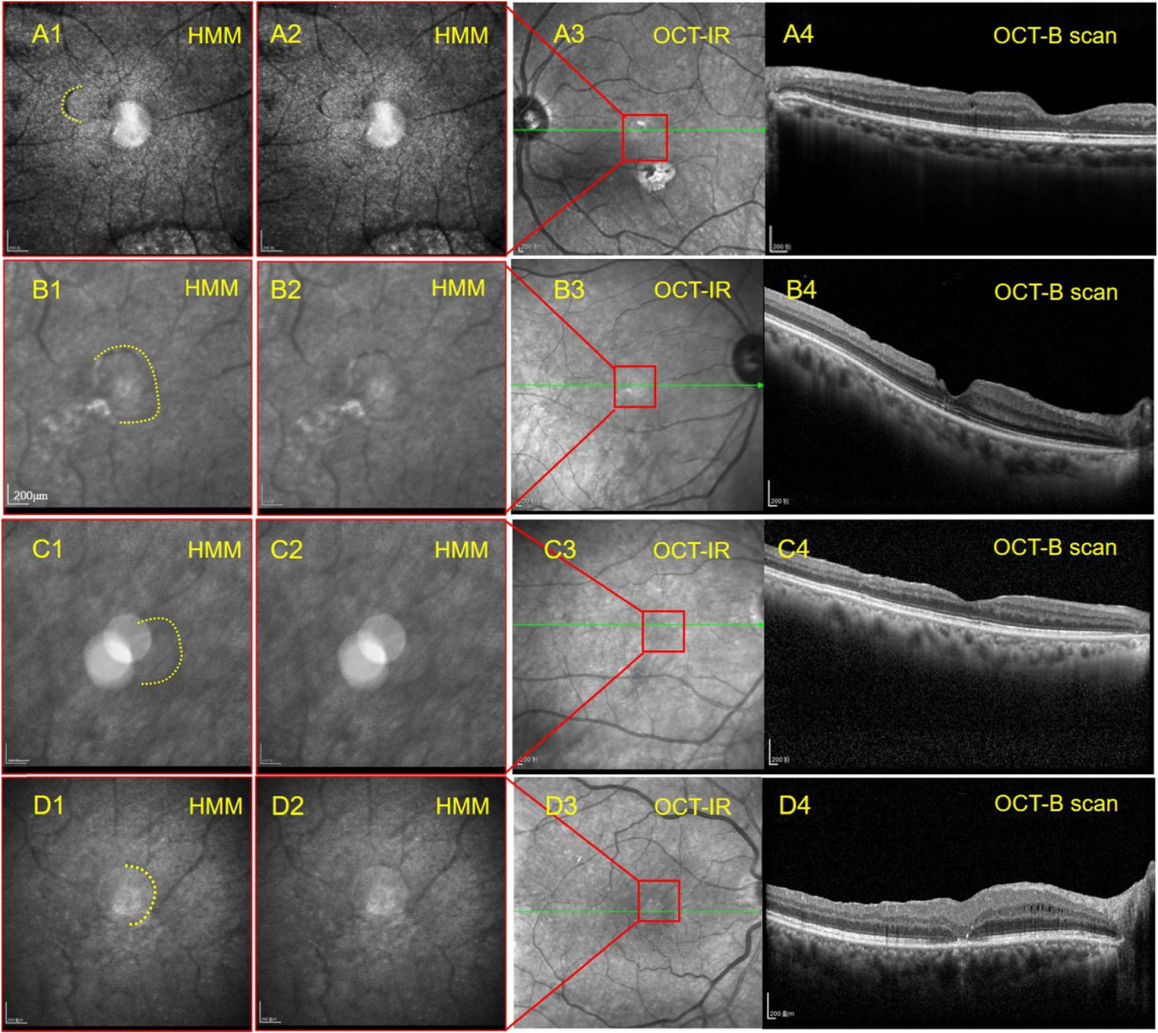
High magnification module (HMM) images, infrared reflectance (IR) and OCT B-scan of 3 completely healed macular hole eyes after surgery (A: case 1, B: case 2, C: case 3) and an eye with an almost completely healed macular hole (D: case 4). A2-D2, 8° × 8° HMM images for macula. There is a dark arc in macula that face optic disc in each of these cases (the dark arc are marked with yellow dotted lines in A1-D1). A3-D3, IR image. A4-D4, OCT-B scan images which showed healed macular hole with integral photoreceptor layer

**Fig. 2:**
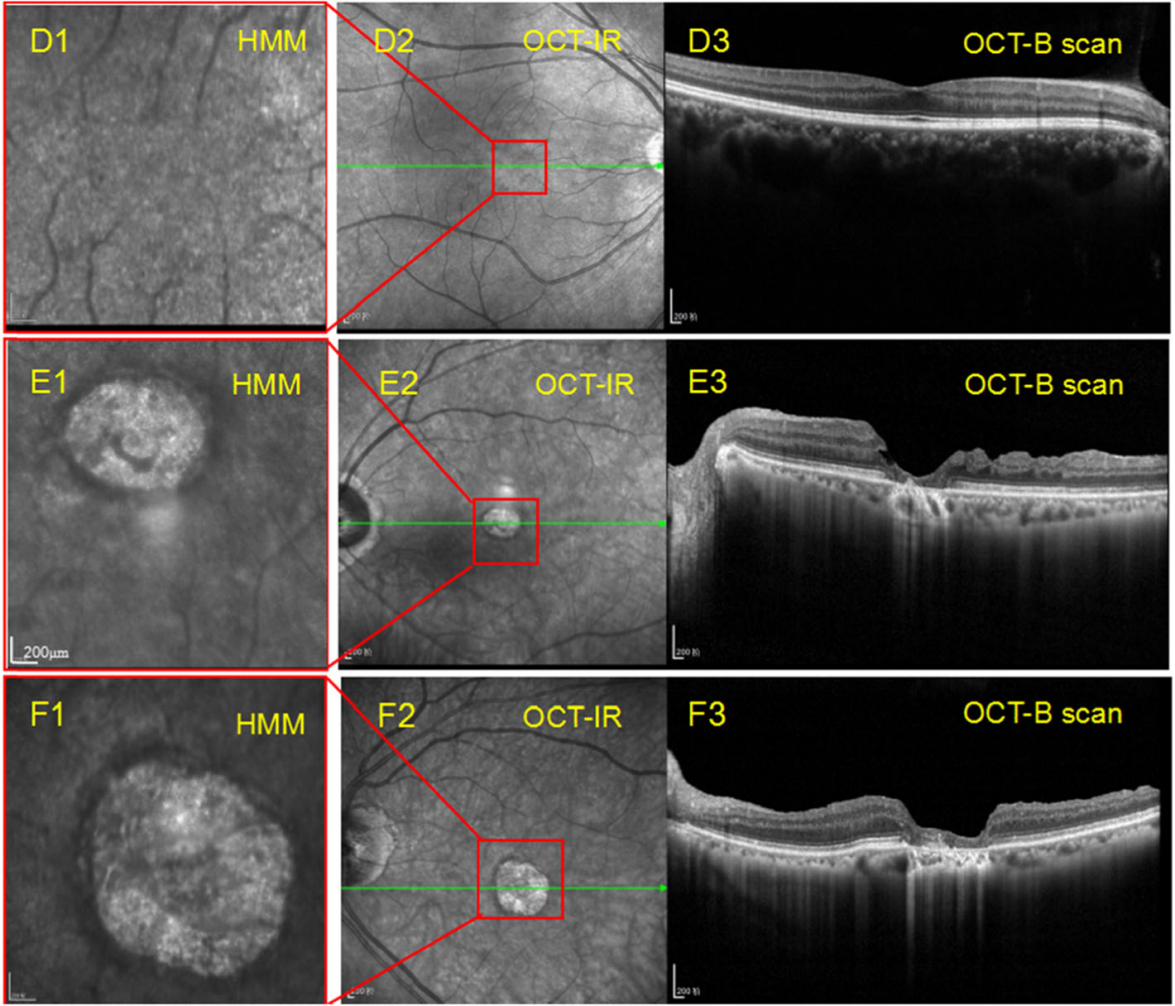
HMM images, IR and OCT B-scan ofa healthy eye and 2 closed macular hole eyes without intact photoreceptor cells layer (D: a healthy eye, E:case 5, F: case 6). D1-F1 8° × 8° HMM images for macula. D1:HMM of health eye display an evenly grey background thickly covered with tiny bright dots in similar size, evenly and widely distributed without dark arc line sign. D2:IR images. D3:OCT B-scan images. D3-OCT-B shows an intact macular layer. E1 and F1 showed a dark ring with bright spots in uneven size inside. E2 and F2 showed IR images. OCT-B of E3 and F3 showed partially closed macular hole lacking of photoreceptor layer

All participants completed examination. OCT B-scan and IR images could be acquired in all of the IMH eyes. All of IMHs have closed as shown in OCT B-scan. Eighteen eyes presented with completely closed macular with intact photoreceptor layer in OCT B-scan and showed better visual acuity. The other 6 eyes presented with closed macular without photoreceptor layer, somewhat like a scar, and showed worse visual acuity (Table 1). Of the 24 IMH eyes, HMM images were obtained in 10 eyes. and 14 eyes yet failed to obtain clear HMM images. HMM images of the healthy eyes (20 fellow eyes of IMH patients and 22 eyes of healthy subjects) displayed an evenly grey background thickly covered with tiny-bright dots in similar size that is evenly and widely distributed (Fig. 2, D1). The shadow of retinal vessels could also be seen. Notably, HMM images of MH eyes showed two kinds of exclusive signs in the fovea area, one is a dark line in arc shape (Fig. 1 A2, B2, C2,D2 for Case1, Case 2, Case 3 and Case 4 respectively. Simultaneously, we outline the dark arc with yellow dotted line in Fig. 1 A1, B1, C1 and D1 respectively.), the other is a dark ring with bright spots inside (Fig. 2 E1, F1 for Case 5, Case6 respectively). In Fig. 1 A1, the arc-shaped dark line was smooth and located at the nasal-superior side of fovea. The arc convex pointed to optic disc and upwardly. In Fig. 1 B1, the arc looked like an inverse C word and the convex pointed to the optic disc, the notch oriented temporally. In Fig. 1 C1, the HMM image was slightly obscure, but we can also find a dark arc pointing to optic disc. In Fig. 1 D1, the HMM image although interfered with a light spot, showed a suspected dark arc with similar characteristics. Whereas, the IR image of macula appeared normal (Fig. 1 A3, B3, C3, D3), and OCT B-can showed that macula closed completely in every single layer including photoreceptor layer (Fig. 1 A4, B4, C4) or nearly complete healing (Fig. 1 D4). HMM image of Case5 and6 (Fig. 2 E1, F1) showed an annular dark line with complex spots inside. The spots were bigger, brighter and in various shape which were different from its peripheral spots. Meanwhile, its IR image showed a similar sign (Fig. 2, E2, F2). OCT B-scan displayed that the macular, although closed, lost its outer layers including photoreceptors cells (Fig. 2, E3, F3). The contralateral eyes of the patients and eyes of healthy subjects had no such changes in HMM images.

## Discussion

Since the first application of OCT in 1991, the technology has constantly made breakthroughs and development. Currently, OCT has been able to show different levels of retina and also make noninvasive dynamic observation and measurement of retinal blood flow. The resolution has raised to 3.9 μm in axial orientation. HMM is a novel module that aimed to investigate the retina at the microstructural level by combining with established OCT multimodal imaging platform. Far from being a simple digital zoom, its resolution has been enhanced to 1.5 μm. Early adopters of this new technology are currently combining the HMM with more established imaging modalities to examine patients with ellipsoid zone disruption and outer retinal changes, early drusen and drusen-like deposits in the context of AMD, and pachychoroid disease, as well as nerve fiber bundle defects (www.HeidelbergEngineering.com). The HMM images reveal details of retinal microstructures that could remain unseen with most imaging modalities. The HMM is FDA-cleared and CE-marked. However, the clinical value has not been widely explored. During our preliminary application of HMM, it was easy to operate and took only a few minutes. HMM images of the healthy eyes displayed an evenly grey background thickly covered with tiny-bright dots that is evenly and widely distributed (Fig. 2 D1). According to the manufacturer, these dots are identified as the photoreceptor cells. Shadow of retinal vessels could also be seen and would cover the dots under the vessels. However, the acquisition rate was low especially in the postoperative eyes.. We failed to acquire clear HMM images in 14 eyes after vitrectomy, whereas all of which had clear IR and OCT images. We have also tried patients with apparent opacity of refractive media, as well as patients without fixed vision, the machine was unable to obtain satisfactory images. The healthy eyes with clear lens and better vision were easy to obtain the HMM images. So high quality HMM images may rely on clear refractive media and good fixation. The HMM system may either not friendly to IOL eyes in terms of our results. Besides, according to the protocol, 1.5 mm pupil size was recommended. The patients’ pupil size was mostly smaller than 2.0–2.5 mm in our study. This might be a reason that we did not acquired clear HMM images in some eyes as larger pupil would bring more aberration [9–11]. Taken together, it seemed that HMM require higher for refractive status, pupil size, fixation, or other condition than traditional OCT.

**Fig. 3:**
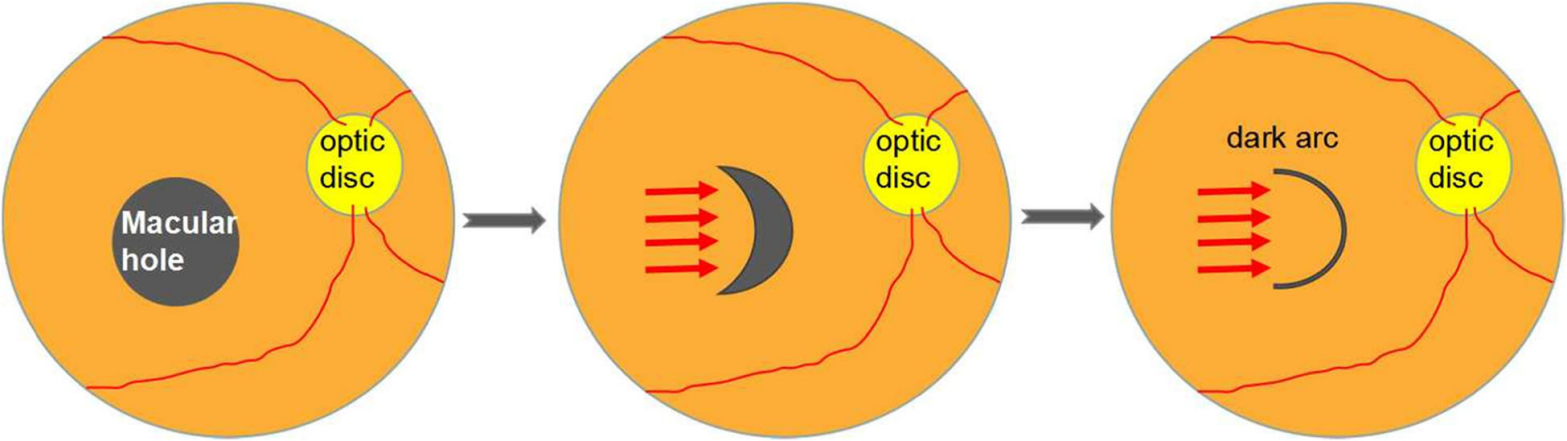
Schematic Diagram depicts a synthesized retina shrank force (red arrows) during the IMH closure process. The dark arc represents the arc line sign located nasally and faced to the optic disc which could be detected in the HMM image of closed IMH eye

To our knowledge, it’s the first time to observe the closed IMH using HMM. Interestingly, new manifestation in HMM images of MH patients’ macula were detected. Four eyes presented similar dark line in an arc shape in HMM images. Meanwhile, their IR images seemed normal and OCT B-scan present perfect or nearly complete closure of macular hole with recovery of outer retina layers including the photoreceptor cells. The arc convex in these eyes are nasal to the fovea and oriented to the optic, and the notch of arc faced temporally. Except for the dark arc the retina displayed grey-white evenly. These arcs somewhat looked like a fissure of outer retina. Considering the ability of detecting microstructure of HMM, we speculated that this dark line displayed here a symbol of macular shrinking and mending mark left after operation. It is interesting, because it was conceived that macula would close centripetally. In such situation, it should be a center of the hole like a dot left at last, not a part of a circle. The arc indicated the macula probably did not close follow a spoke meridian. In other word, the macula was not closed by a completely symmetric centripetal force. Therefore, it was reasonable that temporal retina followed a synthetized vector mainly horizontally towards nasal side to form such an arc. We draw a diagram to depict this presumption. Along with the displacement of temporal retina to nasal side, the macular hole might gradually be squeezed to a crescent moon shaped hole and finally the macula is closed and leaves a fissure like an arc (Fig. 3). Similarly, macular displacement nasally after surgical closure of IMH has been reported in previous studies. The researchers drew the conclusion by comparing the vessels pre-operation with post-operation or the positional relationship to the optic disc [12–21]. Mean displacement distance of vessels was measured in partition sectors around the macula. Every sector displacement contains nasal vector including the nasal sector [18, 19]. However, mean displacement was larger in temporal sector than nasal sector. The final mean displacement was nasal in the whole macula. The retinal displacement in the vertical direction also exited. But it was not identical. Both superior and inferior direction has been reported [18, 19, 21]. Therefore, in fact there must be more complicated vectors for every sector of the retina than shown in the diagram, but if we synthesize each vector, it would mainly orient nasally. The retinal vessels presented displacement of the inner retina whereas, the arc in HMM images reflect the displacement of outer retina. To our knowledge, it is an original observation in this regard.

Besides, we should also consider that whether or not the operation mode would affect the dark arc. Silicone oil or C3F8 filling and the inverted flap technique might affect both anatomy and function of the retina, however, the cases with the dark arc were not filled with silicone oil or C3F8 or treated with inverted flap technique which indicated the dark arc might not be associated with the operation mode.

Case 5 and case 6 presented a dark ring with bright clump or spots inside in the HMM image (Fig. 2 E1, F1). Similar sign was shown in IR image. OCT B-scan indicated that the macula closed with outer retina loss including photoreceptor cell layer. The lack of normal appearance in the fovea in HMM images as the other two MH patients in verse indicates that the HMM image presents the photoreceptor cells. The bright spots in the ring-wrapped zone are different in shape and size from dots outside the ring represents other tissues.

Several mechanisms for MH closure have been suggested: ILM peeling, contraction of the retinal nerve fiber layer (RNFL), and peripheral contraction combined with regional variation of retinal thickness [19]. ILM peeling seemed to induce retinal displacement by removal of the rigid support preventing displacement. And the greater displacement of the temporal retina than the nasal retina toward the optic disc postoperatively suggests that the temporal retina is more flexible and can be retracted toward the optic disc during the MH closure [13]. In addition to ILM peeling, the macular abrasion along the direction of temporal to nasal during the surgery might be of great help [22, 23]. In view of the above mentioned facts, the dark arc might presents “a heal line” of closed macular hole and tend to be located nasally which faces to the optic disc.

This study has many shortcomings. The cases number was limited and we lack of the dynamic observation of macular closure with HMM-OCT. As the device is not equipped with adaptive optics to reduce the optical aberrations, the amount of discernible detail of retina therefore strongly depends on the quality of the patient’s ocular optics, fixation and the technician’s skill. More investigations with HMM are needed to better understand the outer retinal pathophysiology during different stages of IMH and the healing process as well.

**In Conclusion**, the preliminary application of HMM has supplied us a brand-new insight into the microstructure of closed MH. For the first time, we recognized a dark arc in the macular with a convex pointing to the optic disc in HMM images. The dark arc was probably the mark of healed macular after vitrectomy. It indicated the closure of MH may be achieved by displacement of temporal retina to nasal side with a synthesized horizontal force but not symmetric centripetal force. Further studies with a larger number of cases and experimental studies using HMM would be required to confirm our findings and would help to determine the exact mechanism of asymmetric displacement. Furthermore, it called for technology improvement for HMM to achieve wider use in clinic as it seemed that HMM require higher for refractive status, pupil size, fixation, or other condition than traditional OCT.

## Data Availability

The datasets used and/or analyzed during the current study are available from the corresponding author on reasonable request.

## Abbreviations

OCT-HMM: High magnification module (HMM) combining with OCT
IMH: Idiopathic macular hole;
OCT: Optical coherence tomography
BCVA: Best corrected visual acuity
MIVS: Micro-incision vitrectomy surgery
ILM: Internal limiting membrane
C3F8: Perfluoropropane
LogMAR: Logarithm of the minimum angle of resolution
SO: Silicone oil
IR: Infrared reflectance
